# Origins of the change in mechanical strength of silicon/gold nanocomposites during irradiation

**DOI:** 10.1038/s41598-021-98652-y

**Published:** 2021-09-30

**Authors:** Elton Y. Chen, Cameron P. Hopper, Raghuram R. Santhapuram, Rémi Dingreville, Arun K. Nair

**Affiliations:** 1 Center for Integrated Nanotechnologies, Department of Nanostructure Physics, Sandia National Laboratories, Albuquerque, NM 87185 USA; 2 Multiscale Materials Modeling Lab, Department of Mechanical Engineering, University of Arkansas, Fayetteville, AR 72701 USA

**Keywords:** Materials science, Nanoscience and technology

## Abstract

Silicon-based layered nanocomposites, comprised of covalent-metal interfaces, have demonstrated elevated resistance to radiation. The amorphization of the crystalline silicon sublayer during irradiation and/or heating can provide an additional mechanism for accommodating irradiation-induced defects. In this study, we investigated the mechanical strength of irradiated Si-based nanocomposites using atomistic modeling. We first examined dose effects on the defect evolution mechanisms near silicon-gold crystalline and amorphous interfaces. Our simulations reveal the growth of an emergent amorphous interfacial layer with increasing dose, a dominant factor mitigating radiation damage. We then examined the effect of radiation on the mechanical strength of silicon-gold multilayers by constructing yield surfaces. These results demonstrate a rapid onset strength loss with dose. Nearly identical behavior is observed in bulk gold, a phenomenon that can be rooted to the formation of radiation-induced stacking fault tetrahedra which dominate the dislocation emission mechanism during mechanical loading. Taken together, these results advance our understanding of the interaction between radiation-induced point defects and metal-covalent interfaces.

## Introduction

The combination of radiation environments with applied/residual stresses can create a unique and dynamic condition where materials undergo multiple degradation mechanisms simultaneously, potentially compounding and accelerating the undesirable process of aging. Radiation exposure tends to generate nanoscale defects, which can coalesce and form larger defect clusters, dislocation loops, and voids^1^. Most of these defects are detrimental to the material structural integrity and can lead to potential failures such as cracking and fracture with significant dose buildup^2^. There are numerous materials strategies including nanostructuring^3,4^, alloying^5,6^, and interfacial metastable configurations^7,8^ that are currently being investigated to improve radiation resistance. The primary idea behind these developments is to incorporate a high density of defect sinks within the material in order to reduce the accumulation of defects during irradiation^9^. Layered nanocomposites^4^ with silicon (Si) are one such class of novel materials that has demonstrated elevated radiation resistance by leveraging its unique structural characteristics comprised of covalent-metal interfaces^10,11^. Indeed, amorphization of the crystalline Si sublayer^12,13^ during irradiation and/or heating can provide additional defect accommodation opportunities^14^, thus improving the long-term structural stability of such nanocomposites. However, in comparison to the structural characteristics of metallic nanocomposites during irradiation^7,15^, the concurrent deterioration of the mechanical properties is much less understood and studied. Mismatched structural defect concentrations in the sublayers and interfaces can lead to disparate shifts in mechanical performances, affecting the overall composite behavior. By using silicon-gold (Si–Au) as a representative material system, the present study aims to examine the complex relationship between defect accumulation and mechanical performance in these covalent-metal nanocomposites.

Due to the atomistic nature of the defect interactions during radiation, Molecular Dynamics (MD) models have been the preferred tool to study the effects of damage accumulation. For instance, recent studies by^16,]^^17^ have explored the defect generation and accumulation near bimetallic interfaces during low-dose irradiation by simulating the accumulation of displacement cascades. Alternatively, techniques such as Frenkel Pair Accumulation (FPA)^18^ and the Reduced Order Atomistic Cascade (ROAC)^19^ methods have been developed in recent years to facilitate the modeling of high-dose radiation damage within the atomistic timeframe. In our previous works in BCC-BCC and HCP-BCC bimetallic nanocomposites, we used the FPA technique to study both the mechanisms of radiation damage accumulation^7^ and the changes in fracture failure mode^20^ caused by high-dose irradiation. However, Si-based nanocomposites present a different class of interface (covalent-metal) which does not necessarily share the same trait of irradiation-induced degradation mechanisms as bimetallic interfaces. Indeed, previous studies revealed the propensity of Si^12,13^ and similar SiC alloys^21^ to undergo amorphization upon irradiation and heating. Only recently have work by Navale and Demkowicz^22^ begin to explore the radiation tolerance and defect-sink properties for this type of covalent-metal interfaces. Additionally, the mechanical performance of these nanocomposites remains an under-explored subject, with works by several research groups^23,^^24–^^26^, all focusing on a single bulk Si phase. Building upon these foundational studies, the present work aims to take a more holistic approach on the mechanical strength of Si-based nanocomposites during irradiation. By utilizing yield-strength models^23–27^ as an inclusive benchmark, effects of defect accumulation, phase transformation and microstructure evolution can all be captured simultaneously and compared.

We select the Si–Au material system^28^ as a case study of covalent-metal interfacial nanocomposite. Si and Au are immiscible and exhibit limited solubility. This immiscibility allows for Si amorphization during irradiation without causing any unintended growth of an alloying phase. Traditionally grain-on-grain stacking orientation between the nanocomposite layers can be governing factor to its overall microstructure and mechanical properties^7,29^. However, since this type of interface is completely incoherent and due to the rapid insertion of radiation damage and subsequent amorphization of the crystal Si, much of the initial microstructure features can be disregarded in the analysis of dose effects on the mechanical properties of such interfaces. Accordingly, we chose the convenient stacking orientation of $\{001\}/\{001\}$ Si–Au as the bilayer basis of this study. The aligned sublayers also provide the advantage of easily isolating the effects of radiation defects during loading by eliminating any orientation-specific slip activations.

This manuscript is organized as follows. In the “Mechanisms of defect accumulation in Si–Au nanocomposites” section, we first quantify the behaviors of defect accumulation and microstructure evolution within the Si and Au bulk layers. In the “Radiation-induced amorphization” section, we then explore the atomistic processes associated with the Si-based amorphization and interfacial morphology at the Si–Au phase boundaries. In the “Radiation effects on yield surface” section, we present the changes in yield strength associated with the accumulation of radiation damage through the calculation and comparison of yield surfaces. In the “Discussion” section, we explore the origins of the change in mechanical strength due to irradiation. Finally, the procedures for the phase boundary construction and the accelerated-irradiation damage implantation (FPA) are provided in the “Methodology” section.

**Figure 1:**
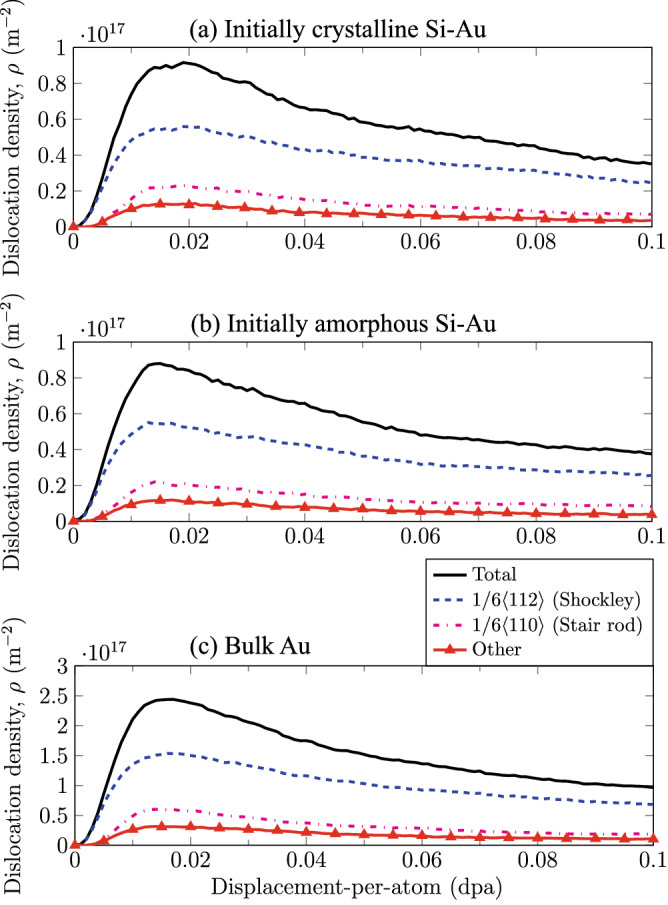
FCC Au dislocation density vs displacement-per-atom in (**a**) Crystalline Si–Au, (**b**) Amorphous Si–Au, and (**c**) Bulk Au. Dislocation density is calculated with respective to total simulation cell. Si–Au bilayers have the volume evenly split between Si and Au.

**Figure 2:**
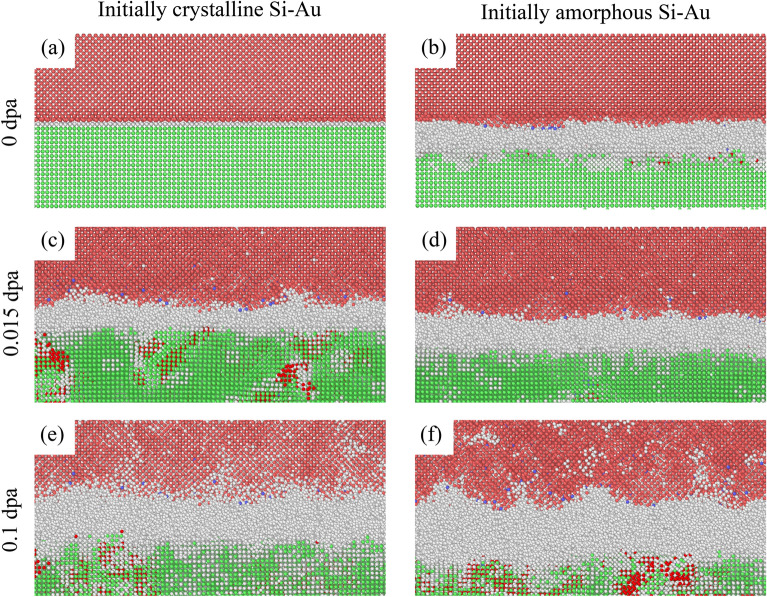
Amorphous Si region growth during irradiation. (**a**, **c**, **e**) $0\,{\mathrm {dpa}}$, $0.015\,{\mathrm {dpa}}$, $0.1\,{\mathrm {dpa}}$ damage level for the initially-crystalline Si–Au nanocomposite. (**b**, **d**, **f**) $0\,{\mathrm {dpa}}$, $0.015\,{\mathrm {dpa}}$, $0.1\,{\mathrm {dpa}}$ damage level for the initially-amorphous Si–Au nanocomposite. Snapshots are rendered at $40\,{\mbox{\normalfont\AA}}$ about the initial interfacial position. Atoms are colored by structure type: White – Amorphous/Defect, Pink – Diamond Si, Green – FCC, Red – HCP, Blue – BCC.

**Figure 3:**
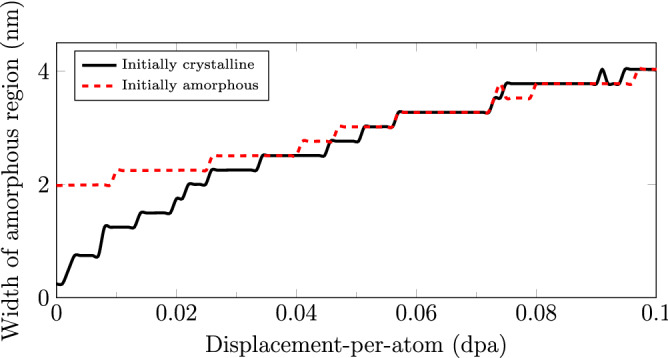
Amorphous layer thickness as a function of damage for the Si–Au nanocomposites. Amorphous region is defined such that more than half of local atoms are identified as defects.

## Results

### Mechanisms of defect accumulation in Si–Au nanocomposites

We begin by examining the microstructure evolution during irradiation in the Si–Au nanocomposites in order to establish a basis for the changes in mechanical performance as a function of the radiation damage. We compare the damage accumulation mechanisms between an initially-crystalline and initially-amorphous Si–Au bilayer. Damage accumulations are simulated up to a damage of $0.1\,{\mathrm {dpa}}$ (displacement-per-atom) for both systems using the FPA technique developed by Chartier et al.^18^. We show the evolution of the dislocation densities as a function of the damage level for both cases in Fig. 1a and b respectively. Note that the damage presented in these sub-figures only represents the FCC Au-rich phase. While some defects can be expected in the Diamond Si-rich phase even before amorphization, our DXA analysis^30^ was unable to identify any significant amount of dislocations in the Si phase. As a reference, the dislocation evolution of an irradiated bulk Au system of similar size is also shown in Fig. 1c, albeit without any grain or phase boundaries present.

Overall, for the initially-crystalline Si–Au, initially-amorphous Si–Au, and bulk Au systems, the process of dislocation accumulation exhibits a similar behavior consisting of an accumulation, a saturation, and amalgamation stages^7^. At first, defects undergo a rapid accumulation of defects at low damage levels, which quickly reaches a saturation peak, after which defects begin to slowly coalesce into larger amalgamated dislocation loops. Saturation peaks in all cases do appear significantly earlier than expected in Au, appearing between 0.015–0.02 dpa. However, the dislocation densities all lay within the expected value for FCC metals, peaking around $9.0 \times 10^{16}\,{\mathrm {m^{-2}}}$ for both bilayer cases and $2.5 \times 10^{17}\,{\mathrm {m^{-2}}}$ for bulk Au. We do not observe any discernible long-term difference in dislocation density or defect accommodation between the two bilayer cases, as amorphization of the interfacial region is always triggered during the irradiation process. However, the absence of the pre-existing amorphous region in the initially-crystalline case does lead to a slightly higher initial peak dislocation density saturation. In contrast to both Si–Au bilayer cases, the dislocation densities in bulk Au is noticeably higher, even when accounting for the $2 \times$ simulation cell volume occupancy. This discrepancy is generally expected and can be attributed to the defect-sink characteristics of the Si–Au phase boundaries, which is absent in the bulk crystal. Finally, unique to FCC metals, we also observe a notably elevated populations of $1/6 \langle 112\rangle$ dislocations. This type of dislocation corresponds to the formation of Stacking Fault Tetrahedras (SFTs) due the clustering of vacancy defects in the lattice, which has been observed both experimentally^31^ and computationally^15,19^. The abundance of SFTs also have significant implication on the mechanical behaviors observed in all systems, as we will later discuss in  “Discussion” section.

### Radiation-induced amorphization

As previously noted, the amorphization of the interfacial regions occurs for both initially-crystalline and initially-amorphous bilayer cases during simulated irradiation. This is because both primary causes of amorphization namely (i) accumulation of defect damages, and (ii) composition mixing of the two immiscible elements (Si and Au) are well-represented by the FPA technique during Frenkel Pair insertion and atomic displacement. To illustrate this effect, the cumulative growths of the primary amorphous region for both crystalline and amorphous cases are shown in Figs. 2 and 3.

While the initially-crystalline system presented in Fig. 2a lacks any inherent amorphous region, we observe a rapid growth of an amorphous region in the vicinity of the interface as demonstrated in Fig. 2e. The final amorphous region thickness for the initially-crystalline case grows up to ${40}{\mbox{\normalfont\AA}}$, doubling that of initially amorphous Si–Au interface. It is important to note that the growth of the amorphous region is nonlinear with respect to the damage level, and plateaus at higher damage levels. Such non-linear behavior reflects the nature of the irradiation-induced composition mixing, which could be approximated as: $C(z,t) = \frac{C'}{2} \left[ 1-{\mathrm {erf}}\left( \frac{z}{\sqrt{\alpha \cdot {\mathrm {\phi }}}} \right) \right]$ ^1,32^, where *z* is the distance normal to the interface plane, $C'$ is the initial bulk concentration, $\alpha$ is the proportionality constant, and ${\mathrm {\phi }}$ is the dose (dpa). Similar growth behavior has also been demonstrated in HCP-BCC with the formation of a third phase in Zr–Nb nanocomposites ^7^. Due to the relative low dose examined in the present study, the concentration gradient do not extend far beyond the interfaces, preserving the overall Si–Au-interface stacking order in the normal direction to the interface.

In contrast, the pre-existing $20\,{\mbox{\normalfont\AA}}$-thick initially-amorphous Si–Au region shown in Fig. 2b and f exhibits a significantly lower growth rate. Interestingly as the dose increases, the thickness seems to double in size and similarly arrive to a thickness of $\sim$ $40\,{\mbox{\normalfont\AA}}$ at $0.1\,{\mathrm {dpa}}$. While $\sim$ $40\,{\mbox{\normalfont\AA}}$ may not be a true stable plateau for the amorphous region thickness, $0.1\,{\mathrm {dpa}}$ is a dose level where we can consider the two bilayer cases converging to a similar behavior. At any point beyond this dose, the initially-amorphous Si region is no longer relevant and the irradiation induced disorder becomes dominant.

### Radiation effects on yield surface

Based on the evolution of dislocation densities in all three cases simulated, we chose four damage levels to evaluate the effect of radiation damage on the mechanical properties:

1. $0\,{\mathrm {dpa}}$ which represents the un-irradiated condition, where the only distinguishing feature between the two interfacial cases is the presence of an initially-amorphous region.
2. $0.005\,{\mathrm {dpa}}$ which represents the early stage of accumulation of defect damage, where the dislocation loops are small in size but the overall dislocation densities are equivalent to those at $0.1\,{\mathrm {dpa}}$.
3. $0.015\,{\mathrm {dpa}}$ which represents the peak of defect accumulation and dislocation density saturation.
4. $0.1\,{\mathrm {dpa}}$ which represents the long-term defect buildup, where dislocation loops have merged to form amalgamated large dislocation networks. Overall defect densities at this damage level are similar to those at $0.005\,{\mathrm {dpa}}$.

The variations in the yield surface from these four cases enable us to differentiate the effects of defect concentration ($0\,{\mathrm {dpa}}$ vs. $0.005\,{\mathrm {dpa}}$ vs. $0.015\,{\mathrm {dpa}}$ vs. $0.1\,{\mathrm {dpa}}$), the nature of the defect content ($0.005\,{\mathrm {dpa}}$ vs. $0.1\,{\mathrm {dpa}}$), and the nature of the interface (crystalline vs. amorphous).

**Figure 4:**
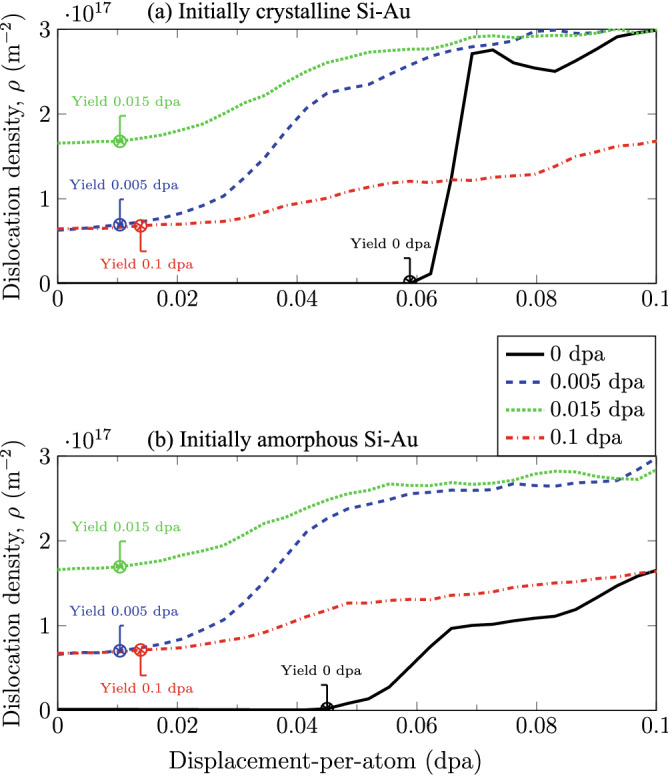
FCC Au dislocation density versus strain $\epsilon$ during biaxial compression at various damage levels. (**a**) Initially-crystalline Si–Au and (**b**) Initially amorphous Si–Au.

In order to extract the various stresses to form the yield surfaces, we first identify the point of yield under strain-controlled loading conditions. We define the point of yield as the point of dislocation nucleation/growth, as compared to the pre-existing defect-content post irradiation prior to any loading. Figure 4 illustrates two examples of the dislocation activities as a function of biaxial-compression strains for various radiation damage levels. By comparing the two $0\,{\mathrm {dpa}}$ curves from the initially-crystalline and the initially-amorphous Si–Au cases, it becomes apparent that the pre-existence of the initial amorphous Si region does indeed affect the dislocation nucleation. Since the un-irradiated initially-crystalline case does not contain any other strain-accommodation mechanism, the strain-induced dislocation nucleation is sharp past the point of yield. In contrast, the un-irradiated initially-amorphous case can partition the strain accommodation between dislocation nucleation and the deformation of the amorphous region. Accordingly, the dislocation nucleation occurs at a lowered strain level. Unfortunately, no clear difference in dislocation activities can be detected between the Si–Au cases post irradiation. This is unexpected since, while amorphization does always occur in both systems, the actual thicknesses of the amorphous region are not congruent with damage level, as shown in Fig. 3. In general, the rates of dislocation growth (i.e., slopes) decrease with increasing damage level. This is attributed to a gradual transition from a nucleation-dominated to a growth-dominated process, such that the creation of new dislocations is fast and sharp, but the extension of existing dislocations is slow and steady. The lower unloaded dislocation densities at $0.1\,{\mathrm {dpa}}$ are also expected from Fig. 1 as a result of dislocation amalgamation at high doses.

**Figure 5:**
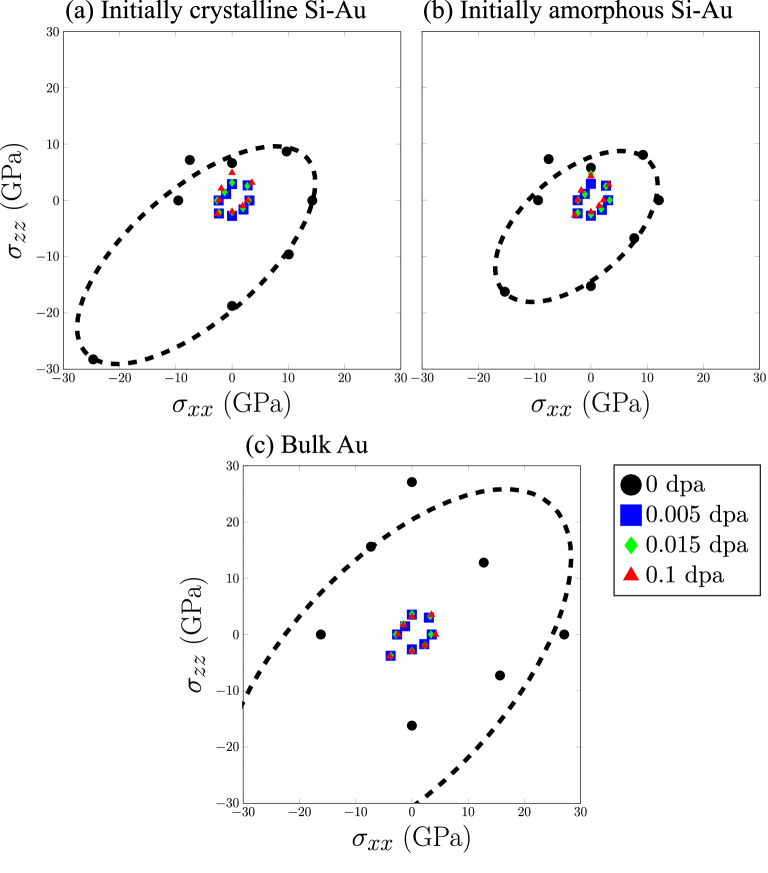
Yield surfaces for (**a**) Initially-crystalline Si–Au, (**b**) Initially amorphous Si–Au, and (**c**) Bulk Au. $\sigma _{xx}$ represents lateral stress. $\sigma _{zz}$ represents interfacial normal stress. Biaxial compression yield point of $0\,{\mathrm {dpa}}$ bulk Au is located at $\sigma _{xx} = \sigma _{zz} =$ $-66.02\,{\mathrm {GPa}}$.

Utilizing the yield points identified based on dislocation activities, we construct the overall yield surfaces in Fig. 5. Figures 5a and b represent the initially-crystalline and initially-amorphous Si–Au cases respectively. As a reference, a third yield surface plotted for the irradiated bulk Au is also presented in Fig. 5c. Expectedly, the introduction of radiation defects into the nanocomposites has a significant impact on its overall mechanical performance. Significant reduction of yield surface can be observed as early as $0.005\,{\mathrm {dpa}}$, where dislocation concentrations are well below half of the peak saturation at $\sim$ $2.5 \times 10^{16}\,{\mathrm {m^{-1}}}$. Further increase in the radiation-damage level also does not appear to have additional influence on mechanical performance, as yield surfaces at $0.015\,{\mathrm {dpa}}$ and $0.1\,{\mathrm {dpa}}$ are largely identical to those at $0.005\,{\mathrm {dpa}}$, within the range of error.

What is less clear is if there exists any correlation between the yield-surface reduction and the presence of the amorphous Si region. An examination of the $0\,{\mathrm {dpa}}$ yield surfaces in Fig. 5a and b shows that the initially-amorphous region does not significantly reduce the yield surface as compared to the one predicted after radiation exposure. The only notable reductions in yield strength appear in the biaxial-compression and uniaxial-compression cases, where the (interfacial) normal yield stress $\sigma _{zz}$ decreases for the initially-amorphous Si–Au interface. As no difference can be observed with tension loading or the lateral yield stress $\sigma _{xx}$, this can be largely concluded as a loss in compressive strength. Alternatively, one may also consider the thickness of the amorphous region, which grows with dose, being an important factor. Nevertheless, this hypothesis can also be disproven. Since the reduction in yield surface is already present at $0.005\,{\mathrm {dpa}}$ for the crystalline case, we simply need to verify the thickness of the newly formed amorphous region. Figure 3 shows that, at this damage level, the thickness is only $\sim$ $7.5\,{\mbox{\normalfont\AA}}$. A value that is significantly less than the initial $20\,{\mbox{\normalfont\AA}}$ region in the $0\,{\mathrm {dpa}}$ initially-amorphous case. Indeed, the overall reduction of yield surfaces can only be attributed to the accumulation of radiation defects. Figure 5c also serves as additional evidence for this conclusion, as the identical dose-dependent yield strength decreases can be observed for bulk Au in absence of any Si or grain boundary.

Interestingly, the matching dose-dependent yield surface reduction between the Si–Au bilayers and the bulk Au reference seems to point to a more fundamental question regarding nanocomposite mechanical performance. What is the governing interfacial component, or “weakest link”? Even without irradiation, we observe that the yield surfaces of the nanocomposite are smaller than the that of bulk Au, particularly with respective to biaxial compression. Such contrast means that either the Si–Au phase boundary or the Diamond Si lattice is the weakest link for yield strength. However, as soon as radiation defects are introduced, the FCC Au sublayer clearly becomes the new weakest component, as bulk Au was able to replicate most of the strength loss. While this dose-dependent deterioration of the yield strength could be common, it may not be universal. Should the nanocomposites be made with Si and another more radiation-resistant metal, the relative importance of Diamond or amorphous Si could increase.

**Figure 6:**
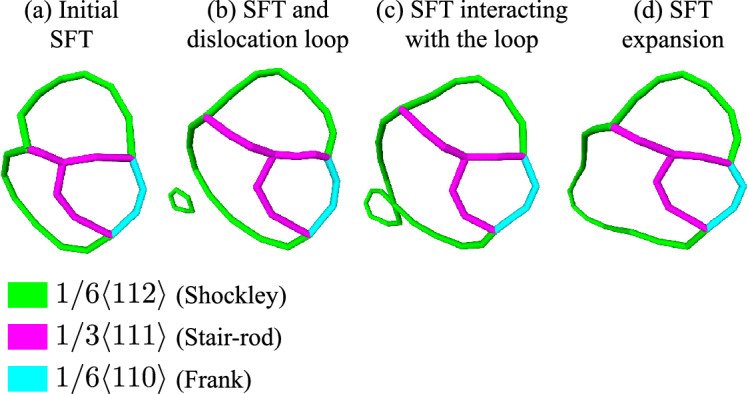
Primary SFT deformation process in irradiated Au. The SFT is isolated from initially-crystalline Si–Au at $0.005\,{\mathrm {dpa}}$ under biaxial compression load.

**Figure 7:**
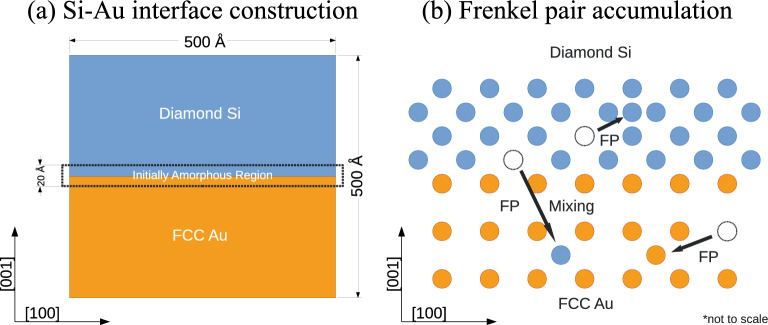
Simulation setup diagrams. (**a**) Construction of the Si–Au nanocomposite with an initial amorphous region. (**b**) Frenkel Pair Accumulation (FPA) schematic.

## Discussion

The rapid softening of the FCC Au phase upon exposure to irradiation observed in this study is an atypical result. While not following conventional understanding of macroscopic irradiation hardening of metals^33^, this nanoscale softening behavior has been observed in other atomistic models of nanoporous Au systems^34,35^ Previous works by Caro and coworkers^34,]^^35^ have demonstrated that the presence of SFTs is the root cause of Au softening. These authors discussed that, under strain loads significantly lower than bulk yield points, SFTs can act as dislocation sources inducing softening, in contrast to the usual behavior in bulk materials, where irradiation-induced defects act as obstacles to dislocation motion, producing hardening. In a closer matching study of CuNb nanocomposites^36^, researchers have also observed irradiation softening and attributed the cause to the presence of SFTs in FCC Cu. Here, the phase boundaries between bulk metallic layers serve the same function as the ligaments seen in nanoporous structures. In all three cases studied in the present work, the emergence of SFTs during irradiation is confirmed by Fig. 1, due to the abundance of characteristic Stair-rod dislocations.

The one important differentiating factor from the aforementioned studies, however, is the relative high defect concentration accumulated as a function of the dose. While this does change the relative order of different load deformation mechanisms, SFTs still play a pivotal role in the overall process. We illustrate in Fig. 6, the early deformation and growth of an SFT in the Au sublayer under biaxial strain of 0.7%. Here, an external dislocation loop approaches a stable SFT, connects with the Shockley partial edges, and expands the SFT volume. As loading continues, SFTs continue to absorb smaller dislocation location loops until all isolated SFTs become connected into a single extended dislocation network resulting from the high concentration of defects. Similar processes of SFT-dislocation interactions have also been modeled under pure thermal diffusion without any mechanical loading^37^. At higher load strains, the formation of the dislocation network further works in conjunction with the aforementioned Shockley-emission mechanism, acting as pre-existing dislocation pathways and lowering the barriers for nucleation. Overall, these changes fundamentally altered the sequence of deformation in irradiated Au crystals, resulting the drop in yield strengths shown in Fig. 5.

Admittedly, these modeling results do lack concrete experimental verifications, but while not necessarily the dominating macroscopic mechanism, irradiation softening is undoubtedly a mechanism that occurs at the nanoscale. Our current hypothesis is that irradiation softening is more prevalent in single crystal metals, which was the case in all of the aforementioned simulation studies^34–37^. However, there are probably other mechanisms and factors at play since experimental studies have shown both hardening^38^ and softening^39,40^ in irradiated single crystals. Another explanation could be related to the radiation condition (i.e. electron vs. neutron irradiation) and the characteristics of the radiation-induced defects. Conventional understanding of radiation hardening stems from a combination of source and friction hardening^1^ processes, both of which require large stationary defects to impede the motion of dislocation in irradiated materials. Such large defects and defect clusters are more characteristic of heavy-ion/neutron irradiation conditions and less common during electron irradiation. This is also match the energetic limitation of common MD radiation models as well as the FPA technique used in the current study. Testing and investigating these competing mechanisms warrant further computational and experimental studies in order to complete our understanding of radiation resistance in covalent/metallic nanocomposites.

## Conclusion

In this study we have examined the changes in mechanical performance of Si-based nanocomposite during irradiation. We compared the performance of an initially-crystalline and an initially-amorphous Si–Au bilayer. Simulated radiation defect accumulation are then performed for each case to accumulate defect damage up to $0.1\,{\mathrm {dpa}}$, followed by mechanical testing to generate the yield surface as a function of irradiation conditions. Our defect analysis shows generally agreeable damage accumulation behaviors amongst the Si–Au nanocomposites and the reference bulk single crystal Au. Decreases in dislocation densities are observed in the nanocomposites and are attributed to phase-boundary defect absorption. Our structural analysis of the nanocomposites reveals the emergence of an amorphous Si–Au region in-between the Diamond Si and FCC Au sublayers, which grows with increasing dose. The constructed yield surfaces demonstrate a rapidly onset strength loss with the introduction of radiation defects. Nearly identical behavior is observed in single crystal bulk Au, leading to the conclusion that FCC Au being the key determining component. The irradiation induced softening behavior in Au is correlated to the formation of SFTs, which dominate the dislocation emission mechanism during mechanical loading. In comparison, the relative effect of amorphous Si–Au presence appears to be trivial as a function of the radiation damage, with all but the minimal influence on interfacial normal compression strength pre-irradiation.

## Methodology

### Atomistic simulation and interatomic potential

We performed all of our Molecular Dynamics simulations with the Large-scale Atomic/Molecular Massively Parallel Simulator (LAMMPS) software package^41^. We selected the Modified Embedded Atom Method (MEAM) potential developed by Ryu et al.^28^ to model the binary Si–Au nanocomposites. MEAM potentials are particularly adapted to model amorphous-Si phase since they can accurately capture the transition from metallic to covalent bonds during the melting process^42^. Since we used the FPA technique to model radiation defect production, we did not need any additional modifications ^43^ to the base interatomic potential. We identified and quantified the defect accumulation and microstructure evolution using the Dislocation Extraction Algorithm (DXA)^30^ as implemented in the OVITO software package^44^.

### Construction of Si–Au covalent-metal interfaces

The schematic describing the workflow related to the interface construction and subsequent irradiation is presented in Fig. 7a. The atomistic construction of a Si–Au bilayer consists of two stacking bulk crystal lattices with periodic boundary conditions applied in all directions. This straightforward construction process results in a nanocomposite with two interfaces, a primary boundary at the stacking surface, and a secondary boundary at the periodic boundary of the simulation cell. As mentioned previously, the present study only considered the basic {100} Si – {100} Au stacking orientation, such that no additional lattice rotation were necessary during initialization. Each simulation cell is constructed to approximately $500\,{\mbox{\normalfont\AA}}\times 500\,{\mbox{\normalfont\AA}}\times 500\,{\mbox{\normalfont\AA}}$ in dimension, with the stacking plane laying normal to the Z-axis. Diamond Si and FCC Au crystal lattices each occupies approximately $250\,{\mbox{\normalfont\AA}}$ of interfacial normal dimension, containing 3,113,640 atoms and 3,690,240 atoms respectively. It is important to note that, due to the periodic boundary conditions, lateral deformations must be applied to both Si and Au to reach commensurability between the lattices. However, these deformations are typically less than one lattice spacing distributed across the superlattice, resulting in negligible maximum strains of $\frac{5.43}{500}$ for Si and $\frac{4.065}{500}$ for Au.

In addition to the initially-crystalline stacking case, we also created an initially-amorphous interfacial configuration. In this case, we heated up a $20\,{\mbox{\normalfont\AA}}$-region centered on the primary interface to 3600 K over a period of 100 ps to induce local melting and atomic mixing in the region. Upon annealing down to ambient temperature of 300 K, the Au-rich region re-solidifies into a FCC lattice with Si segregants; the Si-rich region, however, remains amorphous. Both the crystalline and amorphous systems are thermally relaxed in an isobaric ($P = {0}\ {\hbox {bar}}$) and isothermal ($T =$300 K) NPT ensemble over a period of 200 ps. Final stable atomic configurations are then used as starting configurations for irradiation.

### Simulation of radiation damage: Frenkel pair accumulation (FPA)

In order to simulate the accumulation of radiation defects over a significant dose range, we adopted the Frenkel Pair Accumulation (FPA) ^18,45,46^ technique in lieu of traditional consecutive collision cascades. In contrast to the ROAC technique^19^ which is applicable in dense metallic lattices, only the FPA technique is needed to capture the defect production in the sparse Si lattice, as it impedes compact localized melting^47^. The FPA technique introduces radiation damages directly as interstitial-vacancy point defect (Frenkel) pairs, bypassing the needs to simulate explicit, computationally-expensive collision events. Each Frenkel pair is produced by randomly selecting and displacing an atom from its initial lattice site to a nearby interstitial site $10\,{\mbox{\normalfont\AA}}$ $\sim$ $50\,{\mbox{\normalfont\AA}}$ away. In turn, the initial lattice site becomes a vacancy defect; and the displaced atom becomes an interstitial defect. As the original technique presented by^18^ was developed for ceramic systems with large sparse lattices, it is uniquely-suited to model defect production in the Diamond Si lattice. Schematic of the FPA technique is illustrated in Fig. 7b. Additional optimizations for the dense, metallic systems, such as interstitial site nudging^7^, have also been included for adaptation to FCC Au and amorphous Si regions.

At every 6 ps interval, 6803 randomly generated Frenkel pairs, equivalent to $0.001\,{\mathrm {dpa}}$, are introduced into the nanocomposites. To offset the local energy spike at the new interstitial sites and allow for defect evolutions, each system is subsequently relaxed in a micro-canonical NVE ensemble with an over-dampened Langevin thermostat^48^. The process is repeated for each system until the damage level reaches the level of $0.1\,{\mathrm {dpa}}$, where ${\mathrm {dpa}} = \frac{n_{\mathrm{{fp}}}}{N_{atom}}$. It is important to acknowledge here that the damage rates simulated in the study are several orders of magnitude higher than those achievable by ion irradiation experiments, with each system experiencing approximately ${1.666 \times 10^8}$ dpa/s.

### Calculation of the yield surface

We calculated the yield surfaces^27^ at various defect accumulation levels. We construct individual single yield surface using 8 points consisting of 4 uniaxial tension and compression simulations in the $\langle 100\rangle$ and $\langle 001\rangle$ (interfacial normal) directions, and 4 biaxial tension, compression, tension-compression, and compression-tension simulations about the $\langle 100\rangle$ and $\langle 001\rangle$ directions. Crystallographic and system symmetry deem additional deformation about the secondary lateral $\langle 010\rangle$ direction to be redundant and thus disregarded. In a loading simulation, we deformed the cell in each of the control direction at an engineering strain rate of ${0.001}\,\hbox {ps}^{-1}$. We relaxed the system under an isobaric NPT ensemble with the overall thermostat of $T =$ 300 K and barostat(s) of $P = 0\,\hbox {bar}$ imposed on the remaining uncontrolled directions to freely allow expansion/contraction. We extracted the yield stress at the strain level where significant dislocation activity is detected. For $0\,{\mathrm {dpa}}$ cases, we define dislocation activity as the initial nucleation; for irradiated cases, dislocation activity is defined as the propagation/growth.

## Data Availability

The data that support the findings of this study are available from the corresponding author upon reasonable request.
